# Predator odour but not TMT induces 22-kHz ultrasonic vocalizations in rats that lead to defensive behaviours in conspecifics upon replay

**DOI:** 10.1038/s41598-018-28927-4

**Published:** 2018-07-23

**Authors:** Markus Fendt, Marcel Brosch, Kerstin E. A. Wernecke, Maria Willadsen, Markus Wöhr

**Affiliations:** 1 Institute for Pharmacology and Toxicology, Otto-von-Guericke University Magdeburg, Magdeburg, Germany; 2 Center of Behavioral Brain Sciences (CBBS), Otto-von-Guericke University Magdeburg, Magdeburg, Germany; 3 Integrative Neuroscience Program, Otto-von-Guericke University Magdeburg, Magdeburg, Germany; 4 Behavioral Neuroscience, Experimental and Biological Psychology, Phillips-University of Marburg, Marburg, Germany; 5 Center for Mind, Brain, and Behavior (CMBB), Phillips-University of Marburg, Marburg, Germany

## Abstract

Predator odours induce defensive behaviour in prey animals such as rats. The present study investigated (1) whether laboratory rats exposed to predator odours emit 22-kHz calls which may have an alarming function and (2) whether playback of such calls induces behavioural changes in conspecifics. For this, Sprague-Dawley rats were exposed to samples of fox and lion urine, as well as to the synthetic predator odour TMT. Despite that all odours induced defensive behaviour, only predator urine samples but not TMT were able to induce 22-kHz calls in a few rats. In a second experiment, naive rats were exposed to playback presentations of the 22-kHz calls recorded in the first experiment, as well as to phase-scrambled and frequency-shifted control stimuli. Low intensity playback presentations led to a reduction of locomotor activity during the presentation of the 22-kHz calls but not of the control stimuli. This effect was less specific under high intensity conditions. Taken together the present findings show that natural predator odours are able to induce emission of 22-kHz calls in rats and support the hypothesis that these calls have an alarming function.

## Introduction

For small animals like rodents, defence against predatory threat is a fundamental requirement of life^1^ for often an encounter with a predator ends fatally for the prey^2^. To help to avoid and/or to survive encounters with a predator, rodents – as well as other prey animals – developed very efficient anti-predatory defence strategies, such as freezing^3^. As proposed in Fanselow’s predatory imminence continuum theory^4^, the defensive strategy is dependent on the perceived threat. In rats, only foraging and mating behaviour is typically changed at low risk conditions (pre-encounter stage), whereas at higher risk conditions (post-encounter stage) alterations in freezing or avoidance behaviour can be observed^5–7^. In this stage, also alarm signals can be emitted to warn conspecifics of the presence of predatory threat^8,9^. At the highest level of predatory imminence (circa-strike stage), jumping, fighting, and biting is expressed^4^.

Notably, many of these defensive behaviours are not only elicited by the appearance of predators but also by stimuli predicting a predator such as predator odors^10,11^. In rats, the neuroethology of predator odour-induced defensive behaviours has been extensively studied during the last decades^12–14^. In most studies, the rats were exposed to cat odour, i.e. collars worn by cats^15,16^ or blocks or cloths placed in a cat’s bed or rubbed on the cat’s body^17,18^. However, also odours of other potential predators such as ferrets, minks, foxes, bobcats, wolves, cougars, coyote, and lions were efficient in inducing defensive behaviors^19–23^. Several studies were able to identify single molecules derived from predator odours which are believed to be key components of the odour that triggers defensive behaviours, such as 2,5-dihydro-2,4,5-trimethylthiazoline (TMT) from fox feces^24–26^ or 2-phenyl-ethylamine from carnivore urine^23^.

A very prominent defensive behaviour displayed by rats is the emission of 22-kHz ultrasonic vocalizations^27–29^. These 22-kHz calls are usually relatively long, i.e. between 300 and 3,000 ms, and have a mean peak frequency of approximately 22 kHz, as indicated by their name. Since these calls are emitted in aversive situations, they are believed to reflect a negative emotional state^28^, in order to transmit a potential threat to conspecifics^29^, and/or to warn conspecifics about a potential threat^27^. In many studies, these calls were induced by foot shocks, air puffs, startle stimuli, or drug withdrawal^27–29^. Another approach to elicit 22-kHz calls is to expose rats to a conditioned stimulus (CS), usually a tone, which was previously paired with an unconditioned stimulus (US), typically a foot shock^30^. Surprisingly, there are only very few reports on 22-kHz calls induced by exposure to predators or to predator odours. As shown in the early 1990s by the Blanchards and colleagues^31–33^, exposing rats to a cat efficiently induced the emission of 22-kHz calls. To the best of our knowledge, there is only one published study investigating whether exposure to predator odours can induce 22-kHz calls. In this study, rat lines selectively bred for high and low 50-kHz ultrasonic vocalizations were used^34^, a call type typically occurring in appetitive situations and fulfilling a pro-social function as social contact calls^28,29^. Exposure to a worn cat collar induced low rates of 22-kHz calls in the line selectively bred for high but not low 50-kHz calls, while a substantial number of 22-kHz calls was seen in the random control line^34^. However, there were no odour-free control conditions in this experiment, as well as no detailed analysis of the recorded calls.

The aim of the present study was to characterize the effects of predator odour exposure on the emission of 22-kHz calls in male Sprague-Dawley rats. In our first experiment, rats were exposed to water (control odour), fox urine, lion urine, as well as TMT, with the aim of evaluating which of these odours induce defensive behaviours. The acoustic parameters of the recorded 22-kHz calls were analysed in detail. Our second experiment tested whether the playback of lion urine-induced-22-kHz calls leads to defensive behaviours in experimentally naive rats. To test specificity, fear CS-induced 22-kHz calls were also presented. Finally, to model differences in threat imminence, 22-kHz calls were presented with low and high sound intensity.

## Materials and Methods

### Ethical approval

All experiments were conducted in accordance with the European regulations for animal experiments (2010/63/EU) and approved by the local authorities (University of Magdeburg: Az. 42505-2-1172; University of Marburg: Az. MR 20/35 Nr. 19/2014).

#### Experiment 1 (University of Magdeburg): Predator odour-induced defensive behaviour and ultrasonic vocalizations

Animals and housing: Testing was carried out using 19 experimentally naive male (2–3 months old) Sprague-Dawley rats. Rats were bred and reared at the local animal facility (original breeding stock: Taconic, Denmark). They were housed in groups of 5–6 animals in standard Macrolon Type IV cages (58 cm × 33 cm × 20 cm) with water and standard lab chow (Altromin, Lage, Germany) available ad libitum. Cages were kept in temperature- and humidity-controlled rooms (22 ± 2 °C, 50–55%) with a 12:12 h light/dark cycle (lights on at 6:00 am). All behavioural tests were conducted during the light phase between 10:00 am and 5:00 pm.

Odour samples: 2,3,5-trimethyl-3-thiazoline (TMT) was purchased from PheroTech (Delta, Canada) and fox urine from Maine Outdoor Solutions Inc. (Hermon, ME, USA). Lion urine was obtained from the Zoo of Magdeburg, Germany. All urine samples were aliquoted into 1 ml portions and stored at −18 °C until usage. As a control odour, tap water was used.

Experimental setup and procedure: Testing took place in a standard Macrolon Type III cage (37.5 cm × 22.0 cm × 15.5 cm) covered by an acrylic transparent lid and placed under a fume hood (illumination: ~40 lux). The odour samples were presented in a glass bowl (4 cm diameter, 2.5 cm height), placed, and fixed in the middle of one short side of the test cage. On the opposite side, an ultrasound microphone (for details see below) was positioned outside the test cage next to a hole (diameter: 1.5 cm, height: 6 cm).

On the first day, each rat was singly placed into the test cage for 10 min (without odour sample) to familiarize the rats with the test cage. On the following four days, the odour exposure sessions were performed. First, the odour sample (1 ml of water, fox urine, lion urine, or 5 µl of TMT, respectively) was put into the glass bowl. Second, the experimental rat was gently positioned in the middle of the test cage. Third, the test cage was covered with a transparent acrylic plate to prevent diffusion of the odour through the fume hood. Test duration was 10 min. Each rat was tested once per day and four times in total, with the order of odour samples being counterbalanced within and across days. The test cage was thoroughly cleaned with soapy water after each test and ventilated with clean air.

Recording and analysis of predator odour-induced behavioural changes: Behaviour of the animals was recorded via a video camera mounted above the test cage. Computerized tracking software (EthoVision XT, Version 10, Noldus Information Technologies, Wageningen, The Netherlands) was used to automatically analyse the following behaviours: (a) immobility behaviour (EthoVision software: 2% immobility threshold, averaged over 5 samples), (b) distance travelled, and (c) time spent in odour and no odour area (1/3 of the test cage close and far away from the odour sample, respectively). Furthermore, (d) nose contacts of the animals with the odour sample (number and duration) were manually scored.

Recording and analysis of ultrasonic vocalization: The UltraSoundGate system from Avisoft Bioacustics (Berlin, Germany) was used for recording and analysing ultrasonic signals. For recording, an ultrasound condenser microphone (CM16/CMPA) sensitive to frequencies of 15–180 kHz (flat frequency response between 25 and 140 kHz; ± 6 dB) was used which was connected to a laptop via an USB audio device (UltraSoundGate 116 H). Acoustic data were recorded by AviSoft Recorder USGH software (version 4.2) using a sampling rate of 250,000 Hz in 16-bit format and a recording range of 0–125 kHz.

For offline analysis of the acoustic data, SASLab Pro software (version 5.2) was used. After a fast Fourier transformation (512 FFT length, 100% frame, Hamming window and 75% time window overlap), high resolution spectrograms were produced with a frequency resolution of 488 Hz and a time resolution of 0.512 ms. Onset and offset of the recorded 22 kHz calls were manually marked by a person who was not aware of the experimental condition, and the following parameters were determined and calculated for each single odour exposure session: latency of the first call, number of calls, number of bouts, mean calls per bout, mean call duration, and mean peak frequency. A bout was defined as a call, or a number of calls, separated from other calls by intervals longer than 320 ms^35^.

#### Experiment 2: Playback of predator urine-induced 22-kHz calls (University of Marburg)

Animals and housing: Testing was carried out using 20 experimentally naive male (2–4 months old) Sprague-Dawley rats. Rats were bred and reared at the local animal facility (original breeding stock: Charles River, Germany). They were housed in groups of 3–6 animals in standard Macrolon Type IV cages with high stainless steel covers (58 cm × 33 cm × 20 cm). Water and standard lab chow (Altromin, Lage, Germany) was available ad libitum. Cages were kept in temperature- and humidity-controlled rooms (22 ± 2 °C, 40–70%) with a 12:12 h light/dark cycle (lights on at 6:00 am). All behavioural tests were conducted during the light phase between 8:00 am and 5:00 pm.

Experimental setup and procedure: To test whether lion urine-induced 22-kHz calls induce defensive behaviours in experimentally naive rats, a modified playback protocol previously established was applied^36^. Defensive behaviour was assessed on an elevated radial eight-arm maze (arms: 40.5 × 9.8 cm) under dim red light (~10 lux) conditions^37^.

Acoustic stimuli were presented through an ultrasonic loudspeaker (ScanSpeak, Avisoft Bioacoustics) placed 20 cm away from the end of one arm. An additional, but inactive loudspeaker was arranged symmetrically at the opposite arm as a visual control. Acoustic stimulus presentation was monitored using two ultrasonic condenser microphones (CM16, Avisoft Bioacoustics) placed next to the loudspeakers.

**Figure 1:**
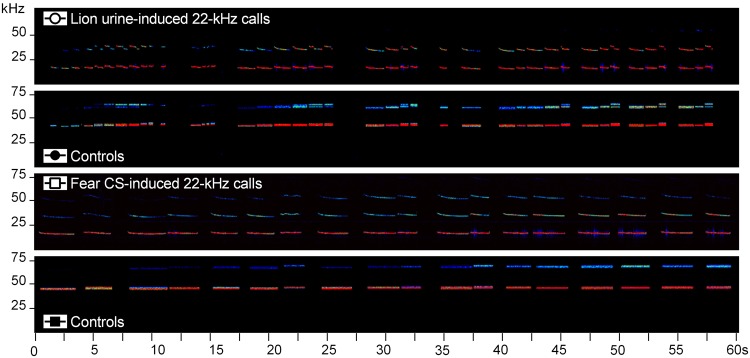
Spectrograms depicting the calls used for playback. The colours code the intensity of the calls (intensity correlates with warmness of the colour).

Four acoustic stimuli were used. This included (I) natural lion urine-induced 22-kHz calls (Fig. 1, first panel) and (II) phase-scrambled and frequency-shifted lion urine-induced 22-kHz controls (Fig. 1, second panel), with the latter serving as a time- and amplitude-matched acoustic stimulus control. To test specificity, this also included (III) natural fear CS-induced 22-kHz calls (Fig. 1, third panel) and (IV) phase-scrambled and frequency-shifted fear CS-induced 22-kHz controls (Fig. 1, forth panel), with the latter again serving as a time- and amplitude-matched acoustic stimulus control. Natural sequences of lion urine-induced and CS-induced 22-kHz calls were chosen to best reflect acoustic features typical for lion urine-induced and CS-induced 22-kHz calls, respectively, under conditions of good signal-to-noise ratios, yet with similar total calling times. Natural lion urine-induced 22-kHz calls and natural fear CS-induced 22-kHz calls were recorded in Magdeburg.

Natural lion urine-induced 22-kHz calls: The natural lion urine-induced 22-kHz calls were recorded from a male Sprague-Dawley rat exposed to lion urine, as described above. The acoustic stimulus contained n = 44 22-kHz calls (total calling time: 37.23 s). Average acoustic call parameters were as follows (mean ± SEM): call duration: 0.84 ± 0.06 s; peak frequency: 19.42 ± 0.11 kHz; downward slope: −0.43 ± 0.12 kHz.

Phase-scrambled and frequency-shifted lion urine-induced 22-kHz controls: The phase-scrambled and frequency-shifted 22-kHz controls were generated with SASLab Pro (Version 4.2, Avisoft Bioacoustics). Specifically, each given 22-kHz call in the original natural lion urine-induced 22-kHz stimulus was first phase-scrambled, i.e. the phase of the original signal was replaced by a random phase. The resulting signal exhibiting the original average power spectrum, but its waveform being a random noise signal, was then shifted up in frequency by 25 kHz, i.e. clearly out of the frequency range 22-kHz calls typically occur^28,29^. The acoustic stimulus contained n = 44 phase-scrambled and frequency-shifted 22-kHz calls (total calling time: 37.23 s). Average acoustic call parameters were as follows (mean ± SEM): call duration: 0.84 ± 0.06 s; peak frequency: 44.42 ± 0.11 kHz; downward slope: 0.00 ± 0.00 kHz.

Natural fear CS-induced 22-kHz calls: The natural CS-induced 22-kHz calls were recorded from a male Sprague-Dawley rat exposed to five fear CS, a 10 kHz tone, in a retention test of a fear conditioning experiment, one day after a fear conditioning session with six pairings of the CS with a 0.8 mA foot shock. The acoustic stimulus contained n = 18 22-kHz calls (total calling time: 43.53 s). Average acoustic call parameters were as follows (mean ± SEM): call duration: 2.42 ± 0.11 s; peak frequency: 21.87 ± 0.13 kHz; downward slope: −3.26 ± 3.08 kHz.

Phase-scrambled and frequency-shifted fear CS-induced 22-kHz controls: The phase-scrambled and frequency-shifted 22-kHz controls were generated with SASLab Pro (Version 4.2, Avisoft Bioacoustics), as described for the phase-scrambled and frequency-shifted lion urine-induced 22-kHz controls. The acoustic stimulus contained n = 18 phase-scrambled and frequency-shifted 22-kHz calls (total calling time: 43.53 s). Average acoustic call parameters were as follows (mean ± SEM): call duration: 2.42 ± 0.11 s; peak frequency: 46.87 ± 0.13 kHz; downward slope: 0.00 ± 0.00 kHz.

All 20 rats were individually exposed to all four acoustic stimuli in counter-balanced order in two subsequent playback sessions separated by two to three weeks. Each session started with an initial 5 min habituation period. Then, the subject rat was exposed to 1 min playback presentations of natural lion urine-induced or CS-induced 22-kHz calls and the respective phase-scrambled and frequency-shifted 22-kHz control separated by a 10 min inter-stimulus interval, followed by a 5 min post-stimulus period.

To model differences in the imminence of threat, all four acoustic stimuli were presented with low and high sound intensity. In a first run with two subsequent sessions (low intensity playback), 22-kHz calls were presented with 40–50 dB SPL. In a second run with two subsequent session two to three weeks later (high intensity playback), 22-kHz calls were presented with 70–80 dB SPL.

Behaviour was monitored by a video camera (Panasonic WV-BP 330/GE, Hamburg, Germany) mounted centrally above the arena. Computerized tracking software (EthoVision XT, Version 10, Noldus Information Technologies, Wageningen, The Netherlands) was used to analyse locomotor activity (distance travelled).

### Descriptive and analytical statistics

Behavioural data are expressed as means ± standard errors of the mean (SEM), whereas acoustic data are shown as whisker box plots. Statistical analyses were performed with GraphPad Prism (version 6.00, GraphPad Software Inc., La Jolla, USA). Data were checked for normal distribution (D’Agostino and Pearson omnibus normality test). Odour or playback effects were analysed by analysis of variance (ANOVA) using odour, area, stimulus, time, and phase as within-subject factors. A p < 0.05 was considered statistically significant.

### Data availability statement

The datasets generated and/or analysed during the current study are available from the corresponding authors on reasonable request.

## Results

### Experiment 1: Predator odour-induced defensive behaviour and ultrasonic vocalizations

#### Defensive behaviours

Our analysis focused on behaviours known to be affected by predator urine samples or TMT^10,25^. Exposure to water was regarded as control condition, i.e. behaviour during exposure to the predator urine samples or TMT was compared with the behaviour during water exposure in post-hoc comparisons.

**Figure 2:**
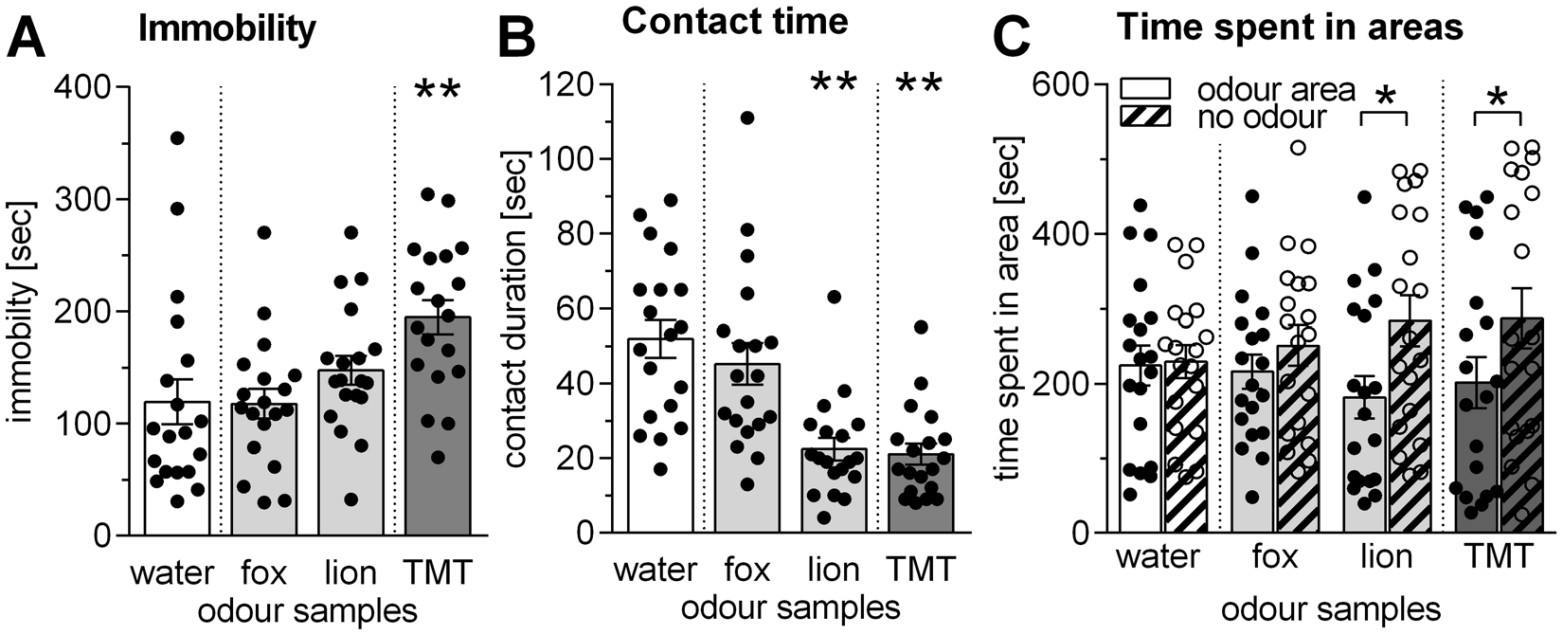
Exposure to predator odour samples induced defensive behaviour in rats. Using a repeated-measure design, rats (n = 19) were exposed to samples of water (control condition), fox urine, lion urine, and TMT. (**A**) Immobility, (**B**) contact time with the odour sample, and (**C**) time spent in the area close to the odour sample (odour area) or far from the odour sample (no odour) was measured and is depicted as means + SEMs. Only exposure to TMT increased immobility of the rats. Both lion urine samples and TMT were significantly less contacted than control samples and rats avoided the area close to these odours. Fox urine samples did not induce behavioural changes in this paradigm. *p < 0.05, **p < 0.01, post-hoc comparison with water or as indicated, after ANOVA.

As shown in Fig. 2A, immobility of the rats was only increased during exposure to TMT [ANOVA: F_3,16_ = 5.78, p = 0.005; post-hoc Dunnett’s test: t_18_ = 4.21, p = 0.002] but not during exposure to samples of fox and lion urine [t_18_ = 0.14, p = 0.99 and t_18_ = 1.58, p = 0.29, respectively]. Furthermore, there was a trend for odour effects on distance travelled (data not shown) [ANOVA: F_3,16_ = 2.73, p = 0.07].

Notably, the contact time with the odour samples was strongly affected by the odours [ANOVA: F_3,16_ = 15.14, p < 0.0001] (Fig. 2B). Post-hoc comparisons revealed a significant decrease of contact time with lion urine samples [t_18_ = 5.13, p = 0.0002] and TMT [t_18_ = 5.77, p < 0.0001] but not with fox urine samples [t_18_ = 1.09, p = 0.57].

Very similar results were obtained if the times spent in the area close to the odour samples (odour area) and the time spent in the area far from the odour sample (no odour area) were compared [ANOVA: F_1,36_ = 4.94; p = 0.03] (Fig. 2C). Post-hoc comparisons showed significantly different area times with lion urine samples [Fisher’s LSD test: t_18_ = 2.59, p = 0.01] and TMT [t_18_ = 2.57, p = 0.01], indicating that these odour samples were avoided. This was not observed with water [t_18_ = 0.26, p = 0.79] or fox urine samples [t_18_ = 0.76, p = 0.45].

#### Ultrasonic vocalizations

**Figure 3:**
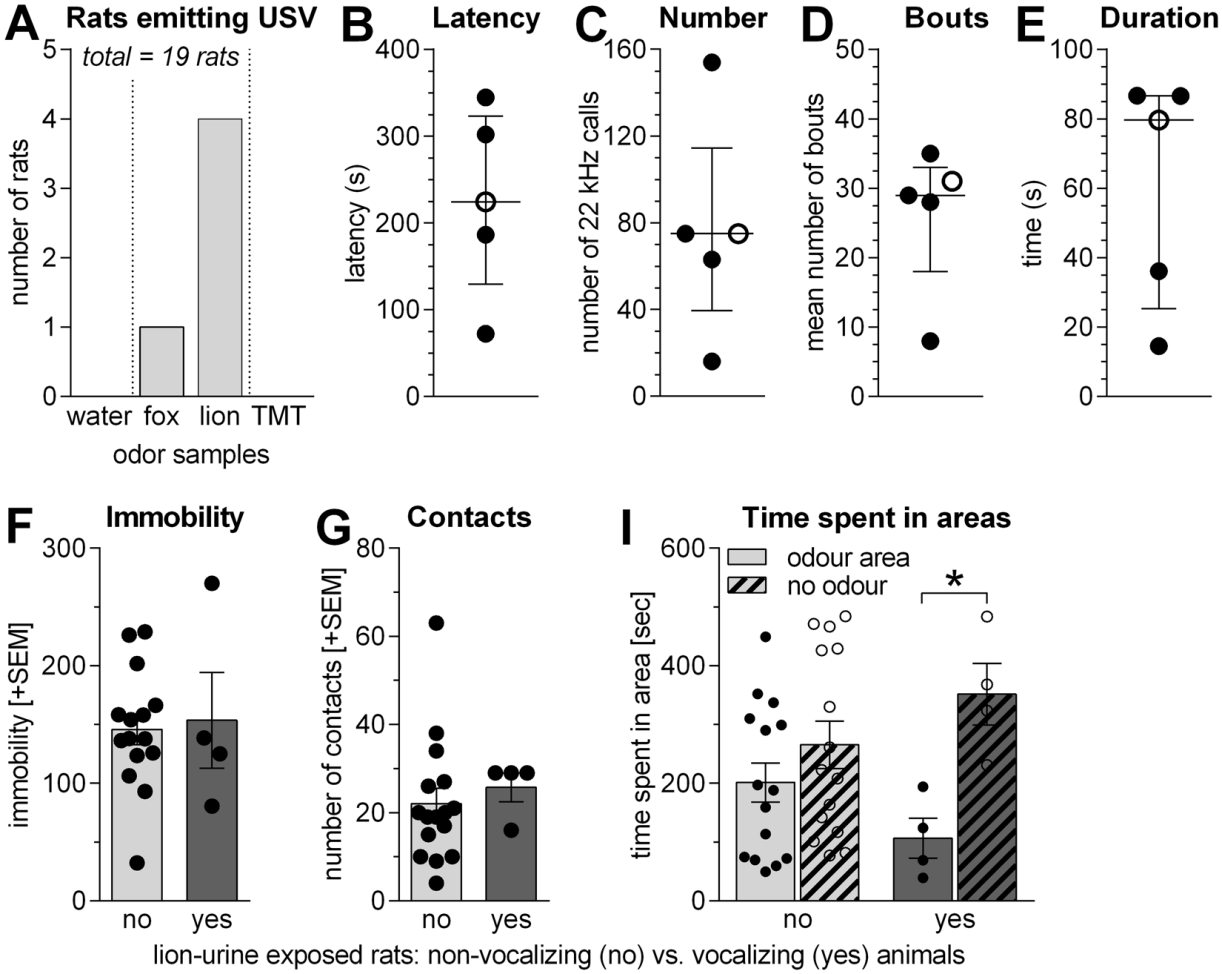
Exposure to predator urine samples induced 22-kHz calls. (**A**) Number of rats emitting 22-kHz calls during exposure to the different odour samples. (**B–E**) Scatter plots depicting the latency, total numbers, numbers of bouts, and total calling time of 22-kH calls for the individual vocalizing rats (open circle = exposure to fox urine; filled circle = exposure to lion urine). The horizontal lines indicate the medians and the interquartile ranges. (**F–I**) Defensive behaviour of non-vocalizing (no) vs. vocalizing (yes) lion urine-exposed rats. The 22-kHz call emitting rats expressed similar immobility (**F**) and contacts with the odour sample (**G**) but more avoidance behaviour (**I**) than the non-vocalizing animals. *p < 0.05, Sidak’s multiple comparison, as indicated, after significant effects in ANOVA.

Figure 3 shows the number of rats emitting 22-kHz calls during testing in different odour exposure conditions. None of the tested rats emitted 22-kHz calls during exposure to water or to TMT. Exposure to the predator samples induced 22-kHz calls in some animals [Chi-square test: χ^2^ = 9.21, df = 3, p = 0.03] (Fig. 3A). Whereas only one rat emitted 22-kHz calls in response to fox urine, four of the 19 rats emitted 22-kHz calls during exposure to lion urine (Fig. 3A). The median latency of the first 22-kHz call was 224 s [range: 72–345 s] (Fig. 3B), the median number of 22-kHz calls was 75 [range: 16–154] (Fig. 3C), the median number of bouts was 29 [range: 8–35] (Fig. 3D), and the median total time spent calling 80 s (range: 15–87 s; Fig. 3E).

We further analysed whether animals that emitted 22 kHz calls express more defensive behaviour than those that did not emit calls. Since only the exposure to lion urine lead to a decent number of vocalizing animals, the analysis was restricted to this condition. Vocalizing and non-vocalizing animals did not differ regarding immobility or contacts with the samples [t-tests: ts < 0.51, p > 0.61] (Fig. 3F,G). However, vocalizing rats spent less time in the odour area than in the no odour area [Sidak’s multiple comparison: t_3_ = 2.57, p = 0.03], an effect which was not observed in non-vocalizing rats [t_14_ = 1.30, p = 0.36; ANOVA: factor area: F_1,34_ = 8.29, p = 0.007; factor group: F_1,34_ = 0.006, p = 0.94; interaction: F_1,34_ = 2.84, p = 0.10] (Fig. 3I).

**Figure 4:**
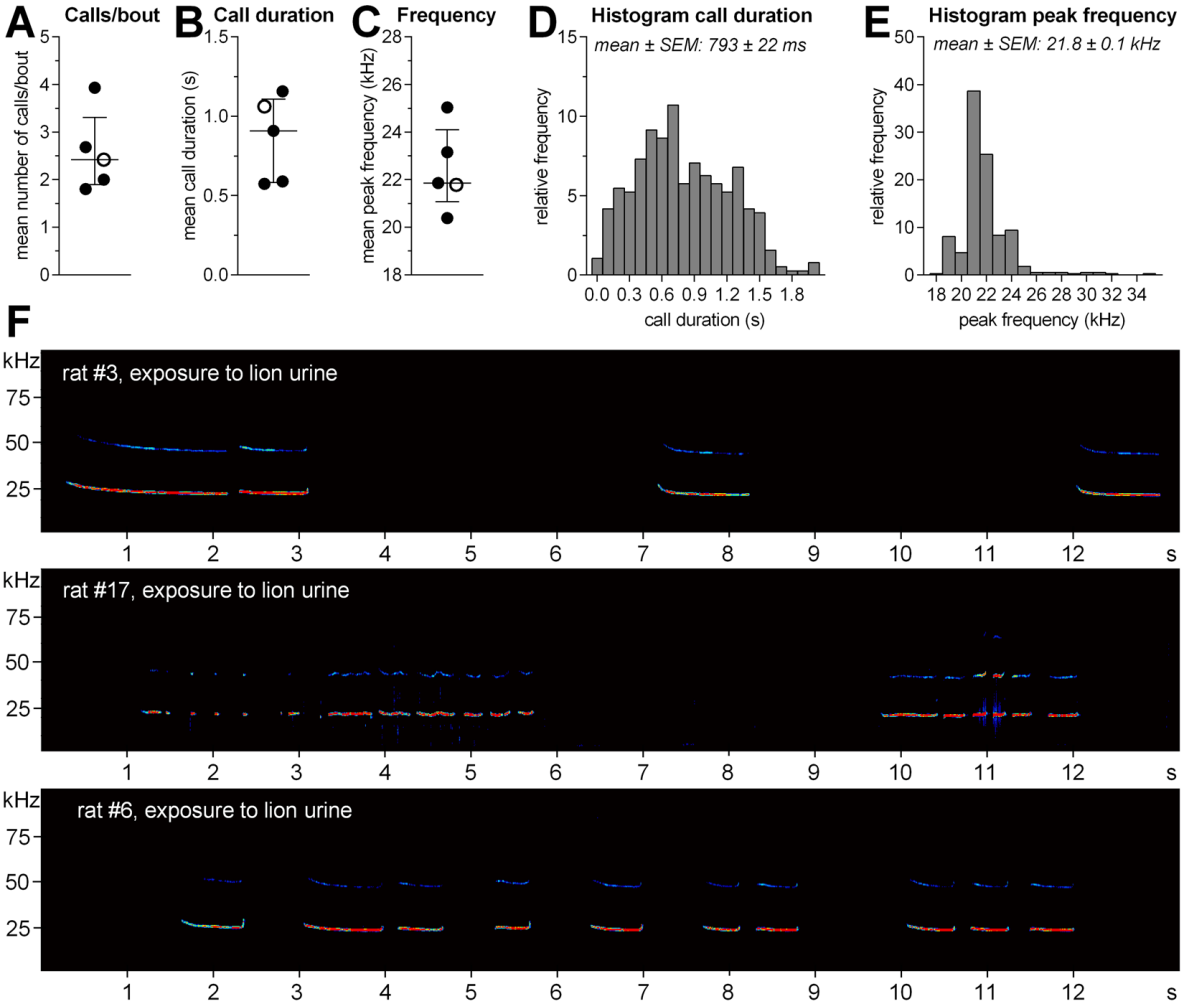
Acoustic parameters of 22-kHz calls induced by exposure to predator urine samples. (**A**) Mean number of calls per bout, (**B**) mean call duration, and (**C**) mean peak frequency of the calls. The horizontal lines indicate the medians and the interquartile ranges (open circle = exposure to fox urine; filled circle = exposure to lion urine). Histograms depicting the distribution of call duration (**D**) and peak frequency (**E**) of all calls recorded during exposure to the different predator urine samples (n = 383), bin widths: 0.2 s and 1 kHz, respectively. (**F**) Exemplary spectrograms showing examples of the recorded 22-kHz calls from three different rats. The colours code the intensity of the calls (intensity correlates with warmness of the colour).

Figure 4 depicts selected acoustic parameters of all recorded 22-kHz calls (n = 383), as well as some exemplary spectrograms of 22-kHz calls from different animals. Note the large variation in the duration of the recorded 22-kHz calls (Fig. 4D–F).

### Experiment 2: Playback of predator urine-induced 22-kHz calls

In this experiment we tested whether playback of lion urine-induced 22-kHz calls (Fig. 1) leads to behavioural changes in naive rats. As control condition, rats were also exposed to playback presentations of 22-kHz calls recorded in a retention test of a fear conditioning experiment (Fig. 1). Rats were individually exposed to all four acoustic stimuli in two subsequent playback sessions. In each session, the rat was exposed to playback presentations of natural lion urine-induced 22-kHz calls (Fig. 1, first panel) or CS-induced 22-kHz calls (Fig. 1, third panel) and the respective phase-scrambled and frequency-shifted 22-kHz control (Fig. 1, second and fourth panel). To model differences in the imminence of threat, all four acoustic stimuli were presented in a first run with low sound intensity (40–50 dB SPL) and then in a second run with high sound intensity (70–80 dB SPL).

**Figure 5:**
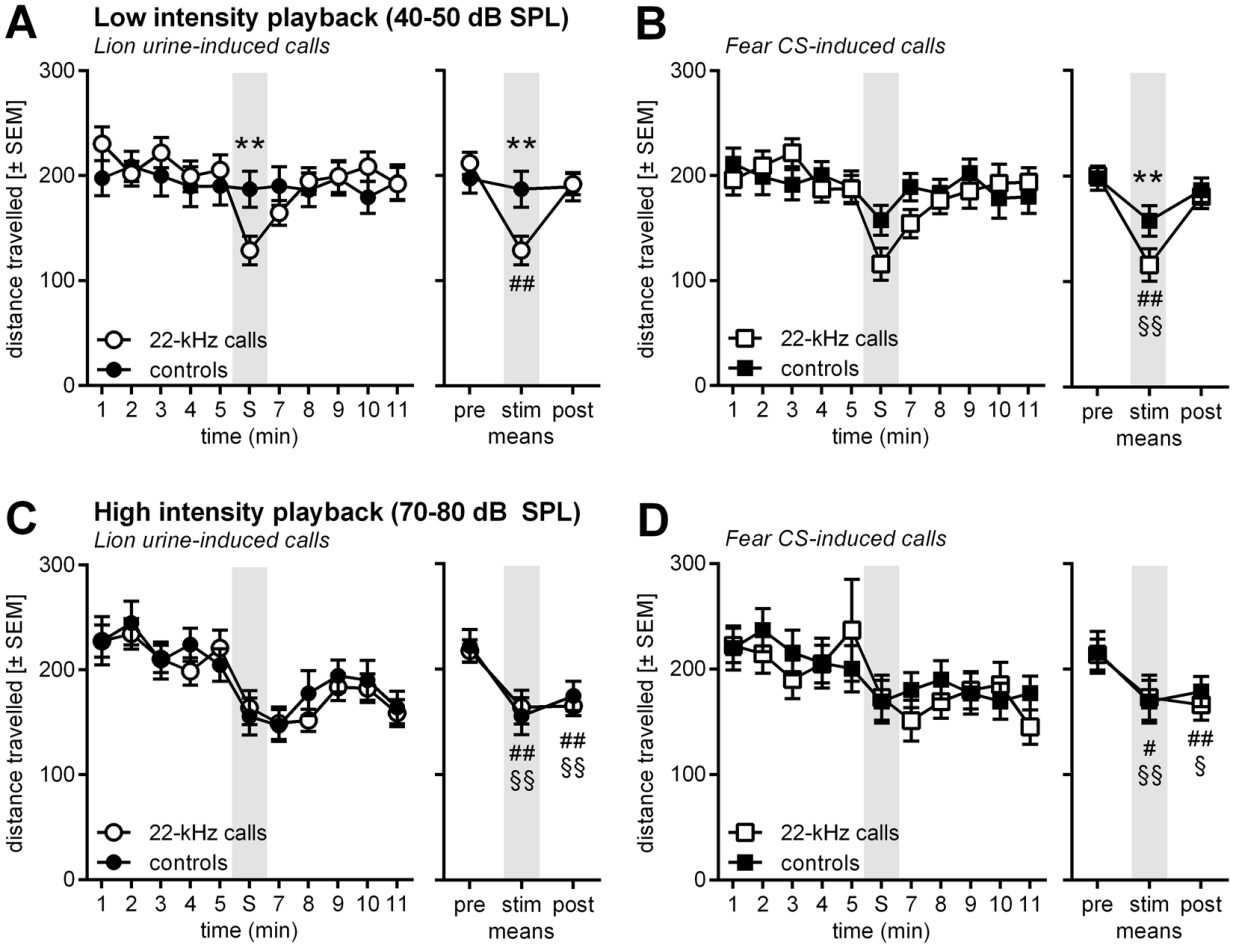
Low intensity playback presentations of lion urine-induced 22-kHz calls or fear CS-induced 22-kHz calls but not of their respective controls led to a reduction in locomotor activity on a radial maze. High intensity playback presentations unspecifically reduced locomotor activity during and after playback. Line diagrams depicting the mean locomotor activity (±SEM) of the rats in 1-min blocks (left panels; S or stim as well as the shaded area indicate the minute of playback presentation) or in the different phases of the experiment (right panel; pre, before playback; stim, during playback; post, after playback presentation). The rats were exposed to playback presentations of low intensity lion urine-induced 22-kHz calls and their respective phase-scrambled and frequency-shifted controls (**A**), low intensity fear CS-induced 22-kHz calls and the respective controls (**B**), high intensity lion urine-induced 22-kHz calls and the respective controls (**C**), and high intensity fear CS-induced 22-kHz calls and the respective controls (**D**). **p < 0.01 (post-hoc Sidak’s comparisons 22-kHz calls vs. control), ^#^p < 0.05, ^##^p < 0.01 (post-hoc Sidak’s comparisons vs. pre-phase; only 22-kHz calls), ^§^p < 0.05, ^§§^p < 0.01 (post-hoc Sidak’s comparisons vs. pre-phase; only controls) after significant effects in ANOVA.

The behaviour most robustly affected by the playback of 22 kHz calls in the present study was distance travelled on the eight-arm maze (Fig. 5A–D). For each of the playback condition, the time course in 1-min blocks (left panels) and the means of the different phases (before, during, and after stimulus playback; right panels) are shown. Notably, playback of lion urine-induced 22-kHz calls with a low intensity but not the respective control induced an reduction of locomotor activity [ANOVA: interaction stimulus x time: F_10,190_ = 2.55, p = 0.007; time: F_10,190_ = 3.74, p = 0.0001; stimulus: F_1,19_ = 0.03, p = 0.86] (Fig. 5A). This reduction was only observed during stimulus presentation [post-hoc Sidak’s comparisons for each minute: t = 3.56, p = 0.005 for stimulus phase; ts < 2.01, ps > 0.40 for all other minutes]. Analysis of the different phases of the test (right panel) supported the previous analysis, i.e. there was a reduction of locomotor activity during presentation of the lion urine-induced 22-kHz calls but not during presentation of the respective control [ANOVA: interaction stimulus x time: F_2,38_ = 9.97, p = 0.003; phase: F_2,38_ = 15.83, p < 0.0001; stimulus: F_1,19_ = 0.79, p = 0.39]. Again, post-hoc Sidak’s comparisons showed significant differences during stimulus presentation [t = 4.71, p < 0.0001] but not in the pre- or post-phase [ts < 1.19, ps > 0.56]. Further comparisons revealed that locomotor activity during playback of lion urine-induced 22-kHz calls was significantly lower than in the pre- and post-phases [ts > 5.10, ps < 0.0001].

Very similar effects were observed during the playback of fear CS-induced 22-kHz calls (Fig. 5B). Time course analysis (left panel) showed that there was a tendency for an attenuation of locomotor activity during playback [ANOVA: interaction stimulus x time: F_10,190_ = 1.70, p = 0.08; time: F_10,190_ = 4.82, p < 0.0001; stimulus: F_1,19_ = 0.25, p = 0.62]. However, analysis of the phases revealed a playback effect [ANOVA: interaction stimulus x time: F_2,38_ = 3.39, p = 0.04; phase: F_2,38_ = 18.64, p < 0.0001; stimulus: F_1,19_ = 1.31, p = 0.27]. Post-hoc tests confirmed a significant difference in locomotor activity during the playback presentations of the fear CS-induced 22-kHz calls and the respective controls [t = 3.27, p = 0.007], as well as during playback of the fear CS-induced 22-kHz calls and the pre- and post-phases [ts > 5.07, ps < 0.0001].

Playback of both types of 22-kHz calls at a higher intensity (Fig. 5C,D) did not induce a specific locomotor activity response when compared with their respective controls [ANOVAs: interactions: Fs < 0.90, ps > 0.53; stimulus: Fs < 0.15, ps > 0.70]. However, there was a strong effect of time [Fs > 4.35, ps < 0.0001]. Post-hoc comparisons showed that locomotor activity was reduced during and after playback of all stimuli at higher intensity, i.e. lion urine induced-22-kHz calls, fear CS-induced 22-kHz calls, and their respective controls [comparison with pre-phase: ts > 2.78, ps < 0.02].

## Discussion

The aim of our first experiment was to investigate defensive behaviours of rats during exposure to predator odours. We used two natural predator odours, samples of fox and lion urine respectively, as well as the synthetic predator odour TMT, a component of fox odor^38,39^. Notably, both natural and synthetic predator odours were able to induce overt defensive behaviours, such as avoidance behaviour. However, a clear dissociation regarding ultrasonic vocalizations was detected. Whereas samples of lion or fox urine induced 22-kHz calls in approximately one fifth of the exposed rats, no single animal emitted 22-kHz calls upon exposure to TMT. In our second experiment, we exposed a different group of rats to playbacks of the 22-kHz calls recorded in the first experiment as well as to fear CS-induced 22-kHz calls. While high intensity playbacks led to unspecific effects, specific effects were seen under low intensity conditions, with 22-kHz calls inducing behavioural inhibition as reflected by a reduction in distance travelled in the radial maze.

Our data support the general finding that both natural and synthetic predator odours are able to induce overt defensive behaviours in rats that are naive to these odors^10,14,25,26^. In the present study, TMT and samples of lion urine induced avoidance behaviour measured by a reduction in contact time with the sample, as well as less time spent in the area in which the sample was presented. Additionally, TMT induced a significant increase in immobility, one of the most prominent behavioural effects of TMT^24,40^. Surprisingly, exposure to fox urine did not induce overt defensive behaviour in the present experiment, although this has been shown in previous studies of our group^20,41–44^ and of others^45–47^. A potential reason for this may be that the experimental protocol of the present study differed from the one used before. However, since we detected robust effects of lion urine samples and TMT, we abstained from optimizing our protocol for fox urine. The robust effects of lion urine are very similar to those published before^23^ and can be explained by the approximately 25 times higher concentration of 2-phenylethylamine in lion urine than in fox urine^23^. Previously, we demonstrated that 2-phenylethylamine is key component of predator urine triggering defensive behavior^23^.

In the present study, exposure to both samples of predator urine induced 22-kHz calls. This is in accordance with the findings of Webber and colleagues^34^ who also demonstrated that exposure to a predator odour is able to induce 22-kHz calls in rats. Whereas Webber and colleagues detected a mean of 8 ± 7 calls in a 5-min exposure session to a cat collar, the present study – if non-calling animals are included in the calculation – revealed a mean of about 18 ± 8 calls in a 10-min exposure session to lion urine. Although it is not explicitly stated in the Webber *et al*. publication, only approximately 30% of the rats in this study emitted 22-kHz calls (H.C. Cromwell, personal communication, September 25, 2017). The low proportion of vocalizing animals in the Webber *et al*. study and in the present study clearly indicate that predator odour does not seem to be a very reliable inducer of 22-kHz calls. This is much less compared with for example fear conditioning studies, in which usually more than half of the rats, sometimes almost all of them, emit 22 kHz calls^30,48^. Nevertheless, the number of emitted calls in the present experiment (70 calls/10 min; Fig. 3) is highly comparable to what has been observed in a fear conditioning experiment with moderate aversive stimuli^30^. In this context, it is important to note that the acoustic parameters of our recorded calls did not differ from 22-kHz calls recorded during fear conditioning^30^, during handling^49^, after air puffs^50^, after acoustic startle stimuli^51^, as well as during cat exposure^33^, indicating the same nature of the calls.

An interesting observation of our study is that exposure to TMT did not induce 22-kHz calls, despite overt defensive behaviour was more pronounced with TMT and that the odour intensity of TMT was much higher than of the predator urine samples. This finding is remarkable in the face of the discussion whether TMT is really perceived by the animals as a predator odour or not^25,52–54^. If TMT has not the ability to induce 22-kHz calls, a well-established species-specific defensive behaviour of rats, this would argue for the idea that TMT does not represent a predator odour. In fact, a recent study raises severe doubts about whether TMT is a component of fox feces^38^, since it was not reliably detectable^55^. Clearly, more studies are necessary to clarify the origin and the properties of TMT.

Our observation that predator odour induced 22-kHz calls leads to the obvious question why rats emit 22-kHz calls during exposure to predator odour or to the predator itself. Notably, the emission of 22-kHz calls is only one of many defensive behaviours in rats and usually not the first that is expressed after encountering a potentially dangerous situation^56–58^. Following the predatory imminence continuum, usually risk-assessment behaviours are first expressed (if the danger is not too immediate), followed by avoidance, escape behaviour (if there are possibilities to do so) or freezing behaviour (if there are no possibilities to hide, avoid, or escape). Ultrasonic vocalization is usually observed after the immediate and active defensive responses^32^, often during freezing behavior^28,49^, with latencies in the minute-range. A potential drawback of 22-kHz call emission might be that these calls are well audible to a substantial number of predators including cats, dogs, and foxes^59^ and thereby guide the attention of the predators to the emitting animal. However, the 22-kHz calls are discussed to have a communicative function, i.e. to serve as alarm calls to warn conspecific about potential danger^28,29^. If they have this function, these calls should be able to affect the emotional state of receiver animals^29^ and thereby also change behaviour of these.

The latter motivated us to perform our second experiment. In this experiment, animals were put on a radial maze and exposed to playback presentations of the lion urine-induced 22-kHz calls recorded in the first experiment. We previously demonstrated that playback presentations of 50-kHz calls but not 22-kHz calls induce approach behaviour, with rats spending more time on the arms of the radial maze next to the loudspeaker^36,37,60^. In the present study, we did not only present the lion urine-induced 22-kHz calls but also 22-kHz calls induced by an auditory fear CS from a fear conditioning experiment. As control stimuli we used phase-scrambled and frequency-shifted versions of the recordings mentioned before. Furthermore, we used two different intensities of the playbacks. The first one was in the 40–50 dB SPL range, the second one in the 70–80 dB SPL range. The high intensity represents the call intensity measured approximately 15 cm away from the call-emitting animal^49^, whereas the low intensity is reached approximately 3–4 m away from a calling rat, a distance which might be normal in a rat pack foraging in nature. Under low intensity conditions, 22-kHz calls (induced by exposure to both lion urine and fear CS) induced a reduction of locomotor activity. This reduction was only observed (1) during but not after playback, and (2) only during playback presentations of the natural 22-kHz calls but not of their respective controls. Although no effects of 22-kHz calls were seen in some studies^61–65^, an attenuation of locomotor activity during or after playback presentations of 22-kHz calls has already been described^36,66,67^. In these studies, noise stimuli, constant sine wave tones, or 50-kHz calls were used as controls. However, in the present study, phase-scrambled and frequency-shifted versions of the natural 22-kHz calls were used as controls. This approach might be more appropriate, particularly because these controls share more acoustic key features with natural 22-kHz calls, particularly total calling time and temporal patterning. The fact that the acoustic control stimuli – at low intensities – did not induce a behavioural response might indicate that rats recognize specific features of the natural calls and do not respond to other similar stimuli in the same way. The difference in behavioural responses between natural 22-kHz calls and their respective controls was most likely driven by the frequency shift. Controls were shifted up in frequency by 25 kHz, i.e. clearly out of the frequency range in which 22-kHz calls typically occur. The effects of phase-scrambling were comparatively mild. This is because 22-kHz calls are typically characterized by very low levels of frequency modulation. The most prominent effect of phase scrambling was that the typical downward slope of 22-kHz calls is completely removed. However, because very little is known about specific acoustic features involved in alarm communication through 22-kHz calls future studies appear warranted. For instance, it would be interesting to present 22-kHz calls originating from several individual rats to link differences in specific acoustic features of the 22-kHz calls between senders to the behavioural response patterns evoked in receivers. Moreover, selective experimental manipulations of individual acoustic features appear of interest. This would also help to rule out the possibility that the behavioural responses observed in the present study are associated with peculiarities in the 22-kHz calls applied here or to the playback treatment in general^68^- although the similarity of the response patterns evoked by natural lion urine-induced 22-kHz calls and fear CS-induced 22-kHz calls clearly speaks for a general effect, particularly when considering the prominent differences in acoustic features between the two stimuli. Notably, the behavioural response to playback presentations was also quite modest, i.e. an attenuation of locomotor activity without avoidance or flight reactions. However, such a response might be adaptive since it helps to identify the actual source of the threat.

With the higher intensity, the behavioural effects of the playback presentations became unspecific, i.e. there was also a response to the respective controls. Furthermore, an attenuation of locomotor activity was not only induced during but also after the presentation of the playback, very similar to the effects described by Brudzynski and Chiu^66^. This unspecific and more pronounced behavioural effect might be adaptive if a potential danger is very close^58^, which might here be indicated by the loudness of the playback.

In summary, the present data demonstrate that rats express overt defensive behaviour and emit 22-kHz calls when exposed to samples of predator urine. TMT only induced overt behaviour but no 22-kHz calls. Playback of the recorded 22-kHz calls attenuated locomotor activity in another group of rats, indicating that these calls are recognized and transmit information.
